# Long-Term Survival After Inter-Hospital Transfer with Extracorporeal Membrane Oxygenation (ECMO): A Retrospective Single-Center Study

**DOI:** 10.3390/jcdd13070337

**Published:** 2026-07-17

**Authors:** Yoganiranjana Dharuman, Sami Sirat, Mirko Doss

**Affiliations:** 1 Department of Cardiothoracic Surgery, University Hospital Aachen, Pauwelsstraße 30, 52074 Aachen, Germany; 2 Department of Cardiothoracic Surgery, Helios Hospital Siegburg, 53721 Siegburg, Germany

**Keywords:** ECMO/ECLS, remote cannulation, mobile health units

## Abstract

Background: Extracorporeal membrane oxygenation (ECMO) is a vital intervention for acute respiratory and cardiac failure. This study evaluates the outcomes, safety, and long-term survival of patients stabilized at external hospitals and transferred to a supra-regional center under ECMO support, comparing results with current global standards. Methods: A retrospective analysis was conducted on 20 patients (14 male, 6 female, mean age 50.6 years) transferred to our hospital. The cohort was divided into veno-venous (vv-ECMO, *n* = 16) and veno-arterial (va-ECMO, *n* = 4) support. Key metrics included weaning success, complication rates, and long-term survival determined via follow-up with a median follow-up of 23 months. Results: Inter-hospital transfer was highly safe; 0% mortality occurred during transport despite a mean distance of 28.7 km (max. 54 km). In the mixed cohort, weaning was successful in 60% of cases, evaluated via 30-day survival. Major complications occurred in eight patients (40%), including bleeding (*n* = 6) and compartment syndrome (*n* = 2). Long-term survival analysis showed that patients who survived the first 30 days had a high probability of continued long-term stability. Conclusions: Remote ECMO cannulation followed by inter-hospital transfer is a safe strategy. While va-ECMO patients face higher mortality due to the underlying severity of cardiac failure, vv-ECMO shows favorable survival rates for ARDS. The specialized “ECMO-retrieval team” model is essential for extending advanced life support to peripheral hospitals.

## 1. Introduction

The clinical utilization of extracorporeal membrane oxygenation (ECMO) and extracorporeal life support (ECLS) has undergone a paradigm shift over the last decade, and the recent ELSO International Report (2025) highlights the necessity of specialized centers for optimal outcomes. Originally developed as a niche therapy for neonates, it has evolved into a cornerstone of modern intensive care medicine for adult patients with refractory respiratory or cardiac failure. The landmark CESAR trial [1] was instrumental in this development, demonstrating a significant survival benefit for patients with severe Acute Respiratory Distress Syndrome (ARDS) when treated at specialized ECMO centers compared to conventional ventilation strategies.

The technical principle of ECMO involves the extracorporeal removal of carbon dioxide and oxygenation of red blood cells via a membrane oxygenator, serving either as a veno-venous (vv-ECMO) support for lung failure or veno-arterial (va-ECMO) support for circulatory collapse. Despite its life-saving potential, ECMO therapy is highly complex, requiring a multidisciplinary team and significant institutional experience to manage the physiological challenges and potential complications, such as major bleeding or systemic inflammatory responses [2,3].

A critical challenge in the provision of ECMO therapy is the regional centralization of specialized centers. Many patients reach the limits of conventional therapy in peripheral or secondary care hospitals that lack the infrastructure for extracorporeal support. To address this, the concept of a “mobile ECMO retrieval team” has been established. This approach involves remote cannulation at the referring hospital followed by inter-hospital transfer while the patient is on active extracorporeal support [4].

While the safety of such transfers has been suggested in early reports, the impact of transport logistics on long-term outcomes and the specific differences between pulmonary and cardiac indications remain areas of active investigation. Most existing registries, such as the ELSO Registry, provide global data, but center-specific analyses are essential to evaluate the efficacy of local “retrieval models.”

This retrospective study aims to evaluate the safety and efficacy of the inter-hospital transfer model implemented at our hospital. We analyzed the clinical course and, most importantly, the long-term survival of 20 patients transferred under ECMO support. By examining both vv-ECMO and va-ECMO cohorts, we seek to identify predictors of survival and provide further evidence for the feasibility of long-distance extracorporeal transport in the critically ill.

## 2. Methods

### 2.1. Study Design and Patient Population

This retrospective single-center study was conducted at our hospital in the department of cardiothoracic and vascular surgery. The study specifically included patients who were cannulated at external hospitals by our specialized retrieval team and subsequently transferred to our center. A total of 20 patients (14 male, 6 female) were identified and categorized into two groups based on the mode of support: veno-venous (vv-ECMO, *n* = 16) for respiratory failure and veno-arterial (va-ECMO, *n* = 4) for cardiac or circulatory failure.

### 2.2. Our Retrieval Model

Upon request from a peripheral hospital, a specialized team consisting of a cardiac surgeon, a perfusionist, and an anesthesiologist was dispatched. Remote cannulation was performed on-site using the Seldinger technique. Patients were stabilized and then transferred via ground ambulance or helicopter, depending on the distance and urgency. Throughout the transfer, continuous monitoring of vital parameters and ECMO function (flow, sweep gas, pressures) was maintained, following established protocols for high-risk transport [5].

### 2.3. Data Collection and Clinical Parameters

Patient data were retrospectively extracted from MetaVision (Version 6, iMDsoft, Düsseldorf, Germany) and Orbis (Agfa HealthCare GmbH, Bonn, Germany). For patients transferred before recovery, discharge summaries from external hospitals were obtained.

Statistical analysis was limited to descriptive statistics due to the small cohort size (*n* = 20) and the exploratory nature of the study. Continuous variables are presented as means ± standard deviation (SD). Categorical variables are expressed as absolute frequencies (*n*) and percentages (%) of the total sample. No inferential statistics, survival analyses, or regression models were performed. All descriptive data processing and chart generation were conducted using Microsoft Excel (Microsoft Corp., Redmond, WA, USA). Key parameters included ECMO indication, transport distance, complications, and duration of therapy. Outcomes were defined as 30-day survival, hospital discharge, and long-term survival.

### 2.4. Definitions and Follow-Up

“Weaning success” was defined as the successful explantation of the ECMO system without the need for re-cannulation within 48 h. The primary endpoint was 30-day survival. Long-term survival was assessed through hospital records and follow-up inquiries with a median observation period of 23 months. Complications were recorded based on the ELSO definitions [6].

## 3. Results

### 3.1. Patient Demographics and Baseline Characteristics

A total of 20 patients were included in the study, with a mean age of 50.6 ± 16.8 years (range 16–82 years). The cohort was predominantly male (70%).

The primary indication for veno-venous support is ARDS, accounting for 81% of cases. Secondary indications like TRALI and re-warming are less frequent at 6% each (Table 1). Cardiogenic shock due to three-vessel coronary artery disease (CAD) represents the main indication (50%) for VA-ECMO. Post-CPR failure and septic shock with cardiac involvement each constitute 25% of the indications (Table 2).

### 3.2. Transfer Safety

The retrieval team performed cannulations at 24 different external hospitals. The mean transport distance was 28.7 km (range 10 km–54.1 km). All 20 transfers were completed without any technical failures or patient fatalities during transit (0% transport mortality).

### 3.3. Clinical Course and Survival Outcomes

The clinical efficacy of the ECMO support was evaluated based on weaning success and short-term survival. Successful weaning and explantation from the ECMO system were achieved in 12 of the 20 patients (60%). This success rate directly correlates with the observed 30-day survival rate of 60%. Conversely, mortality during the extracorporeal support phase was 40% (Table 3).

The intensity and complexity of the treatment are further reflected in the duration of support. The mean duration of therapy was 10.35 ± 8.9 days, indicating a significant period of mechanical assistance required for pulmonary or circulatory recovery. Long-term follow-up data suggest that for patients who successfully survived the initial 30-day period, the probability of continued survival remained high. This indicates that the most critical clinical period is concentrated within the first month following cannulation.

### 3.4. Complications

The clinical evaluation showed that 8 out of 20 patients (40%) experienced complications during ECMO support (Table 4).

- Hemorrhagic events were the most frequent complication, affecting six patients (30%).
- These events included diffuse bleeding in three cases, intracranial hemorrhage (ICB) in two cases, and one instance of hemothorax.
- Vascular complications occurred in two patients (10%), presenting as compartment syndrome of the lower leg (*n* = 1) and the abdomen (*n* = 1).
- Regarding mortality, the overall rate was 40% (*n* = 8), with multi-organ failure (MOF) being the leading cause of death (*n* = 3).

## 4. Discussion

The present study demonstrates that the inter-hospital transfer of critically ill patients supported by extracorporeal membrane oxygenation (ECMO) is a safe and feasible strategy. Our findings of 0% transport mortality across 20 cases align with specialized retrieval programs worldwide and are consistent with recent data from Liu et al. [7], confirming that the risks associated with transport are secondary to the risks of the underlying disease when managed by an expert team [8].

### 4.1. Survival and Benchmarking 

The observed 30-day survival rate of 60% in our cohort is consistent with the results of the landmark CESAR trial [1], which demonstrated the efficacy of ECMO in specialized centers. Furthermore, our results align with the findings of Noah et al. [9], who reported similar survival rates for critically ill patients requiring specialized retrieval and extracorporeal support during the H1N1 pandemic. These results underline the safety and effectiveness of our model.

### 4.2. Complications and Organ Failure

Complications occurred in eight patients (40%) during ECMO therapy. The complication rate in our cohort is consistent with international data reported by Brogan et al. [10], who identified bleeding as the most frequent complication during ECMO support in the ELSO registry. The occurrence of bleeding complications in six cases (30%) highlights the ongoing challenge of balancing systemic anticoagulation with the risks of hemorrhage in patients with severe coagulopathy [11].

### 4.3. Long-Term Survival and Clinical Implications

Long-term survival was assessed via a median follow-up of 23 months, reflecting current research trends in Health-Related Quality of Life (HRQOL) [12]. Interestingly, our long-term follow-up indicates that the survival curve stabilizes significantly after the first 30 days. This suggests that if a patient can be successfully weaned and survives the initial critical phase, the long-term prognosis is favorable. This “bridge to recovery” potential justifies the logistical effort and costs associated with mobile ECMO teams.

Our model proves that distance is not a limiting factor for ECMO therapy. Stabilization on-site at the referring hospital prevents further clinical deterioration during transport. We argue that early contact with a specialized center and prompt initiation of extracorporeal support are more decisive for the outcome than the actual transport time.

During the follow-up period after hospital discharge, one patient in our study died 22 months after ECMO therapy due to complications resulting from paraplegia. Consequently, the long-term survival rate in our cohort was 50%.

To date, few studies focus specifically on long-term survival following inter-hospital transfer on ECMO, as most investigations utilize 30-day survival as their primary endpoint. Our results align with the findings of Starck et al. [13], who observed that patients successfully weaned from ECMO (67%) remained alive after a mean follow-up of 374 days. With a mean follow-up of 23 months, our study offers a significantly longer observation period, providing valuable insights into the extended post-clinical course.

Recent data further validate these findings. Previous reports [14] reported one-year survival rates for veno-arterial ECMO between 40% and 50%, which is highly consistent with our observed outcomes.

Furthermore, current ELSO Registry analyses by Biscotti et al. [15] confirm that specialized inter-hospital transfer (mobile ECMO) does not carry a higher mortality risk compared to stationary treatment in a tertiary center. Finally, studies by Schmidt et al. [16] on long-term outcomes show that survival often reaches a stable plateau after 6 to 12 months. Our 50% survival rate after nearly two years underlines that the initial effort of inter-hospital transfer translates into a sustainable long-term benefit for this critically ill patient population.

When interpreting our findings, the single-center design and the specific cohort size of 20 patients (16 V-V and 4 V-A cases) provide a focused framework. This setting highlights the precise operational dynamics of a dedicated local retrieval program, complementing large-scale, multi-center experiences such as those reported by Lucchini et al. [17], Mihu et al. [18], and Sams et al. [19]. Furthermore, while extensive epidemiological registries, such as the landmark cohorts published by Rossong et al. [20], Cho et al. [21], and Treml et al. [22], are designed to analyze broad long-term survival, predictors of mortality, or overall quality of life across thousands of patients, our study intentionally prioritizes the acute safety, logistical precision, and clinical outcomes achieved during the initial transport and retrieval phase. Consequently, our findings offer highly detailed, consecutive, real-world insights that serve as a valuable and practical addition to the wider scientific literature.

## 5. Limitations

This study is limited by its retrospective design and the relatively small sample size of 20 patients. Furthermore, as a single-center study, the results may be influenced by local protocols and the specific patient demographics.

## 6. Conclusions

The inter-hospital transfer of ECMO patients by a specialized retrieval team is safe and effective. While mortality remains high, particularly in va-ECMO for cariogenic shock, the survival rates for respiratory failure support the continued use of this life-saving intervention. Future studies should focus on refining patient selection criteria to further optimize long-term survival.

## Figures and Tables

**Table 1:** Indications for veno-venous support (V-V ECMO).

Indication	Percentage (%)
ARDS (Acute Respiratory Distress Syndrome)	81% (*n* = 13)
TRALI (Transfusion-Related Acute Lung Injury)	6% (*n* = 1)
Re-warming	6% (*n* = 1)
MODS in Sepsis (Multiple Organ Dysfunction Syndrome)	6% (*n* = 1)

**Table 2:** Indications for veno-arterial support (V-A ECMO).

Indication	Percentage (%)
Cardiogenic shock in three-vessel CAD	50% (*n* = 2)
Cardiopulmonary failure after resuscitation (CPR)	25% (*n* = 1)
Septic shock with cardiopulmonary failure	25% (*n* = 1)

**Table 3:** ECMO outcomes and therapy duration.

Clinical Outcome	Total (*n* = 20)
Successful Weaning/Explantation	60.0% (*n* = 12)
30-Day Survival Rate	60.0% (*n* = 12)
Overall Mortality	40.0% (*n* = 8)
Mean Duration of Therapy	10.35 ± 8.9 days

**Table 4:** Overview of ECMO-related complications.

Complication	Total (*n* = 20)
**Bleeding (Total)**	6 (30%)
Diffuse Bleeding	3 (15%)
Intracranial (ICB)	2 (10%)
Hemothorax	1 (5%)
**Compartment Syndrome**	2 (10%)
Lower Leg	1 (5%)
Abdomen	1 (5%)

## Data Availability

The data presented in this study are available on request from the corresponding author (the data are not publicly available due to privacy or ethical restrictions.).

## Abbreviations

ARDS	Acute Respiratory Distress Syndrome
CAD	Coronary Artery Disease
CESAR	Conventional Ventilation or ECMO for Severe Adult Respiratory Failure (Trial)
CPR	Cardiopulmonary Resuscitation
ECLS	Extracorporeal Life Support
ELSO	Extracorporeal Life Support Organization
H1N1	Hemagglutinin Type 1 Neuraminidase Type 1 (Influenza A Virus)
HRQOL	Health-Related Quality of Life
ICB	Intracranial Bleeding
MODS	Multiple Organ Dysfunction Syndrome
MOF	Multiple Organ Failure
TRALI	Transfusion-Related Acute Lung Injury
va-ECMO	Veno-arterial Extracorporeal Membrane Oxygenation
vv-ECMO	Veno-venous Extracorporeal Membrane Oxygenation

## References

[1] Peek G.J., Mugford M., Tiruvoipati R., Wilson A., Allen E., Thalanany M.M., Hibbert C.L., Truesdale A., Clemens F., Cooper N. (2009). Efficacy and economic assessment of conventional ventilatory support versus extracorporeal membrane oxygenation for severe adult respiratory failure (CESAR): A multicentre randomised controlled trial. Lancet.

[2] Brodie D., Bacchetta M. (2011). Extracorporeal membrane oxygenation for ARDS in adults. N. Engl. J. Med..

[3] Combes A., Bacchetta M., Brodie D., Müller T., Pellegrino V. (2012). Extracorporeal membrane oxygenation for respiratory failure in adults. Curr. Opin. Crit. Care.

[4] Ehrentraut S.F., Schroll B., Lenkeit S., Ehrentraut H., Bode C., Kreyer S., Kögl F., Lehmann F., Muders T., Scholz M. (2019). Interprofessional two-man team approach for interhospital transport of ARDS-patients under extracorporeal membrane oxygenation: A 10 years retrospective observational cohort study. BMC Anesthesiol..

[5] Heuer J.F., Mirschel M., Bleckmann A., Quintel M., Moerer O. (2019). Interhospital transport of ARDS patients on extracorporeal membrane oxygenation. J. Artif. Organs.

[6] Tonna J.E., Abrams D., Brodie D., Greenwood J.C., Rubio Mateo-Sidron J.A., Usman A., Fan E. (2021). Management of Adult Patients Supported with Venovenous Extracorporeal Membrane Oxygenation (VV ECMO): Guideline from the Extracorporeal Life Support Organization (ELSO). ASAIO J..

[7] Liu L., Hu D., Hao T., Chen S., Chen L., Zhu Y., Jin C., Wu J., Fu H., Qiu H. (2024). Outcomes and risk factors of transported patients with extracorporeal membrane oxygenation: An ECMO center experience. J. Intensive Med..

[8] Mendes P.V., de Albuquerque Gallo C., Besen B.A.M.P., Hirota A.S., de Oliveira Nardi R., Dos Santos E.V., Li H.Y., Joelsons D., Costa E.L.V., Foronda F.K. (2017). Transportation of patients on extracorporeal membrane oxygenation: A tertiary medical center experience and systematic review of the literature. Ann. Intensive Care.

[9] Noah M.A., Peek G.J., Finney S.J., Griffiths M.J., Harrison D.A., Grieve R., Sadique M.Z., Sekhon J.S., McAuley D.F., Firmin R.K. (2011). Referral to an extracorporeal membrane oxygenation center and mortality among patients with severe 2009 influenza A(H1N1). JAMA.

[10] Brogan T.V., Thiagarajan R.R., Rycus P.T., Bartlett R.H., Bratton S.L. (2009). Extracorporeal membrane oxygenation in adults with severe respiratory failure: A multi-center database. Intensive Care Med..

[11] Beiderlinden M., Treschan T., Görlinger K., Peters J. (2007). Argatroban in extracorporeal membrane oxygenation. Artif. Organs.

[12] Jolink F.E.J., Onrust M., Dos Reis Miranda D., Mandigers L., Delnoij T., Maas J.J., Raasveld S.J., Vlaar A.P.J., Donker D.W., Scholten E. (2025). Dutch Extracorporeal Life Support (ECLS) Study Group. Five Years After Extracorporeal Membrane Oxygenation: A Prospective Cohort Study of Health-Related Quality of Life and Patient Outcomes. Crit. Care Med..

[13] Starck C.T., Hasenclever P., Falk V., Wilhelm M.J. (2013). Interhospital transfer of seriously sick ARDS patients using veno-venous Extracorporeal Membrane Oxygenation (ECMO): Concept of an ECMO transport team. Int. J. Crit. Illn. Inj. Sci..

[14] Lorusso R., Raffa G.M., Alenizy K., Sluijpers N., Makhoul M., Brodie D., McMullan M., Wang I.W., Meani P., MacLaren G. (2019). Structured review of post-cardiotomy extracorporeal membrane oxygenation: Part 1-Adult patients. J. Heart Lung Transplant..

[15] Biscotti M., Agerstrand C., Abrams D., Ginsburg M., Sonett J., Mongero L., Takayama H., Brodie D., Bacchetta M. (2015). One Hundred Transports on Extracorporeal Support to an Extracorporeal Membrane Oxygenation Center. Ann. Thorac. Surg..

[16] Schmidt M., Bailey M., Sheldrake J., Hodgson C., Aubron C., Rycus P.T., Scheinkestel C., Cooper D.J., Brodie D., Pellegrino V. (2014). Predicting survival after extracorporeal membrane oxygenation for severe acute respiratory failure. The Respiratory Extracorporeal Membrane Oxygenation Survival Prediction (RESP) score. Am. J. Respir. Crit. Care Med..

[17] Lucchini A., Gariboldi R., Villa M., Cannizzo L., Pegoraro F., Fumagalli L., Rona R., Foti G., Giani M. (2023). ASST Monza—Mobile ECMO team group. One hundred ECMO retrivals before and during the Covid-19 pandemic: An observational study. Intensive Crit. Care Nurs..

[18] Mihu M.R., Maybauer M.O., Cain K., Swant L.V., Harper M.D., Schoaps R.S., Brewer J.M., Sharif A., Benson C., El Banayosy A.M. (2023). Bridging the gap: Safety and outcomes of intensivist-led ECMO retrievals. Front. Med..

[19] Sams V.G., Anderson J., Hunninghake J., Gonzales M. (2022). Adult ECMO in the En Route Care Environment: Overview and Practical Considerations of Managing ECMO Patients During Transport. Curr. Trauma. Rep..

[20] Rossong H., Debreuil S., Yan W., Hiebert B.M., Singal R.K., Arora R.C., Yamashita M.H. (2023). Long-term survival and quality of life after extracorporeal membrane oxygenation. J. Thorac. Cardiovasc. Surg..

[21] Cho H.W., Song I.A., Oh T.K. (2021). Quality of Life and Long-Term Mortality Among Survivors of Extracorporeal Membrane Oxygenation: A Nationwide Cohort Study in South Korea. Crit. Care Med..

[22] Treml B., Breitkopf R., Bukumirić Z., Bachler M., Boesch J., Rajsic S. (2022). ECMO Predictors of Mortality: A 10-Year Referral Centre Experience. J. Clin. Med..

